# Supplementary material for: Associations between vigorous physical activity, social ties, social support, and self-reported health among older adults in Accra, Ghana
Source: PLOS Glob Public Health. 2023 Feb 13;3(2):e0001582. doi: 10.1371/journal.pgph.0001582 (PMC10021291; doi:10.1371/journal.pgph.0001582)
Supplement: S1 Appendix — (DOC) [file pgph.0001582.s001.doc]

**S1 Appendix (Questionnaire)**

**DEMOGRAPHIC AND PERSONAL CHARACTERISTICS OF PARTICIPANTS**

1. What is your gender?

Male [ ] Female [ ]

1. What is your highest educational level?

Basic education [ ]

Secondary education [ ]

HND/Diploma [ ]

First degree [ ]

Master’s degree [ ]

PhD or equivalent [ ]

Other (specify) ……………………………………………

1. Which of the following age groups do you belong to?

55-64 yrs [ ]

65-74 yrs [ ]

75-84 yrs [ ]

85-94 yrs [ ]

95-104 yrs [ ]

Above 104 yrs [ ]

1. In which of the following categories does your net monthly income fall? All amounts are in Ghana Cedis.

0-200 [ ]

201-400 [ ]

401-600 [ ]

601-800 [ ]

801-1,000 [ ]

Above 1,000 [ ]

1. How much do you spend a day?

Less than Ghc50 [ ] Ghc50-100 [ ] More than Ghc100 [ ]

1. What is your marital status?

Single [ ] Divorced [ ] Widowed [ ] Married [ ]

1. Which of the following cardiovascular diseases do you have? Choose ‘none’ if you don’t have any.

Hypertension [ ]

Diabetes [ ]

Stroke [ ]

Any heart disease [ ]

Others (specify) …………………………..

None [ ]

1. What is your employment status? Not employed [ ] Employed [ ]
2. How many children do you have?

None [ ] 1-5 [ ] Above 5 [ ]

**SOCIAL TIES**

1. How many siblings (brothers and sisters) do you have?

None [ ] 1-5 [ ] Above 5 [ ]

1. How many **OTHER BLOOD RELATIONS** (i.e. uncles, aunties, cousins, nephews, nieces, etc.) do you have?

None [ ] 1-5 [ ] Above 5 [ ]

1. How many **DEPENDANTS** do you have?

None [ ] 1-5 [ ] Above 5 [ ]

1. How many **CLOSE AND MUTUAL** friends, workmates, and other acquaintances do you have?

None [ ] 1-5 [ ] Above 5 [ ]

1. How often have you voluntarily participated in social and/or community intervention programs (e.g. communal labour, advocacy, social campaign, etc.)?

Never [ ] Less often [ ] Very often [ ]

**SOCIAL SUPPORT**

1. How many blood relations, friends and/or workmates will readily extend social support to you when you need it?

None [ ] 1-5 [ ] Above 5 [ ]

**VIGOROUS PHYSICAL ACTIVITY**

1. How much time do you spend on vigorous physical activity such as running in a typical day?

Not at all [ ]

Less than 30 minutes [ ]

30-60 minutes [ ]

61-90 minutes [ ]

91-120 minutes [ ]

121-150 minutes [ ]

Above 150 minutes [ ]

**SELF-REPORTED HEALTH**

1. How would you rate your health? Poor [ ] Good [ ]

End of Survey! Thank You
