# Supplementary material for: Associations between vigorous physical activity, social ties, social support, and self-reported health among older adults in Accra, Ghana
Source: PLOS Glob Public Health. 2023 Feb 13;3(2):e0001582. doi: 10.1371/journal.pgph.0001582 (PMC10021291; doi:10.1371/journal.pgph.0001582)
Supplement: S2 Appendix — (DOC) [file pgph.0001582.s002.doc]

S2 Appendix. Variable categorisation, definition, and coding

| # | Indicators | Definition | Coding |
| --- | --- | --- | --- |
| 1 | Children | Number of children the individual had | None - 1; 1-5 - 2; Above 5 - 3 |
| 2 | Siblings | Number of brothers and sisters the individual had | None - 1; 1-5 - 2; Above 5 - 3 |
| 3 | Other blood relations | Number of other blood relations (apart from siblings and children) the individual had | None - 1; 1-5 - 2; Above 5 - 3 |
| 4 | Dependants | Number of individuals being cared and fend for by the individual | None - 1; 1-5 - 2; Above 5 - 3 |
| 5 | Close and mutual friends | Number of close and mutual friends (including workmates) the individual had | None - 1; 1-5 - 2; Above 5 - 3 |
| 6 | Social support | The number of individuals who could readily provide financial, emotional, psychological and other forms of support to the individual | None - 1; 1-5 - 2; Above 5 - 3 |
| 7 | Self-reported health | Whether the individual perceived his or her health to be good or poor | Poor - 0; Good - 1 |
| 8 | Vigorous PA | Time spent in vigorous physical activities such as running in a typical day | <30 – 0; ≥ 30 – 1 |
| 9 | Gender | The sex of the individual | Male - 1; female - 2 |
| 10 | Education | The highest education of the individual | Basic - 1; secondary -2; tertiary -3 |
| 11 | Age | The age (in years) of the person | 60-64 -1; ≥65 - 2 |
| 12 | Income | The net income (in cedis) of the individual | ≤200 - 1; 201-400 - 2; ≥401 - 3 |
| 13 | Marital status | Whether the individual was married or not | Not married - 1; Married - 2 |
| 14 | Employment status | Whether the individual was employed or not | Not employed – 1; Employed - 2 |
