# Supplementary material for: Associations between vigorous physical activity, social ties, social support, and self-reported health among older adults in Accra, Ghana
Source: PLOS Glob Public Health. 2023 Feb 13;3(2):e0001582. doi: 10.1371/journal.pgph.0001582 (PMC10021291; doi:10.1371/journal.pgph.0001582)
Supplement: Attachment — Submitted filename: Response to Editors.doc [file pgph.0001582.s004.doc]

**Response to Editors and Reviewers**

**General response**: We thank the reviewers and editors for making time to review our manuscript despite their busy schedule and COVID-19 disruptions. We’ve found their suggestions helpful; we are happy to revise our manuscript with your expert suggestions. We’ve highlighted our response to each comment in yellow. The changes are also tracked in the manuscript.

Comments

1. Does this manuscript meet PLOS Global Public Health’s publication criteria? Is the manuscript technically sound, and do the data support the conclusions? The manuscript must describe methodologically and ethically rigorous research with conclusions that are appropriately drawn based on the data presented.

Reviewer #1: Yes

Reviewer #2: Yes

2. Has the statistical analysis been performed appropriately and rigorously?

Reviewer #1: Yes

Reviewer #2: Yes

3. Have the authors made all data underlying the findings in their manuscript fully available (please refer to the Data Availability Statement at the start of the manuscript PDF file)?

The PLOS Data policy requires authors to make all data underlying the findings described in their manuscript fully available without restriction, with rare exception. The data should be provided as part of the manuscript or its supporting information, or deposited to a public repository. For example, in addition to summary statistics, the data points behind means, medians and variance measures should be available. If there are restrictions on publicly sharing data—e.g. participant privacy or use of data from a third party—those must be specified.

Reviewer #1: Yes

Reviewer #2: No

4. Is the manuscript presented in an intelligible fashion and written in standard English?

PLOS Global Public Health does not copyedit accepted manuscripts, so the language in submitted articles must be clear, correct, and unambiguous. Any typographical or grammatical errors should be corrected at revision, so please note any specific errors here.

Reviewer #1: Yes

Reviewer #2: Yes

5. Review Comments to the Author

Please use the space provided to explain your answers to the questions above. You may also include additional comments for the author, including concerns about dual publication, research ethics, or publication ethics. (Please upload your review as an attachment if it exceeds 20,000 characters)

Reviewer #1: Congratulations to the research team. This study appears to be very thoughtful with sound analytical basis which produced findings which collaborates existing evidence as well as provide new insights into vigorous physical activity and self-reported health impacts among older adults especially relating to social ties and social supports from children and siblings or blood relations. The public health implications as articulated in the conclusion are relevant for the improvement of health and wellbeing relating emanating from physical activity being it moderate or vigorous. Congratulations.

**Our response**: Thank you very much; we appreciate your time and support.

Reviewer #2: This is an interesting study, given that we are moving to an aging population that needs support in terms of improving their health outcome. I have the following comments that need to be considered by the author:

**Our response**: We really value your expert opinions, and we have improved the paper based on each comment. Our response to each comment is given below.

**Comment**: 1. It is not clear which institution or organization provided ethical approval for the study.

**Our response**: This is true, we have mentioned the institution and country where it was based.

**Comment: 2**. Table 2: Chi square sign is missing, p value = 0.000 should be replaced with p < 0.001

**Our response**: Well noted; we have effected the changes.

**Comment:** 3. Authors claim to have done adjusted Odd Ratios (ORs); however, table 3 does not show results for the adjusted odds ratio

**Our response**: the ORs in the table (Table 3) are adjusted odd ratios; anytime logistic regression captures confounding variables alongside the primary predictors, the associated odd ratios are automatically adjusted.

**Comment:** 4. Study limitation is missing, given that the population studied are elderly people who accessed Social Security and National Insurance Trust (SSNIT) in Accra, what about others who did not and maybe those who are not covered by the insurance trust.

**Our response**: We agree with you. Thanks for this; we’ve revised the limitation section under discussions.
