# Supplementary material for: Associations between vigorous physical activity, social ties, social support, and self-reported health among older adults in Accra, Ghana
Source: PLOS Glob Public Health. 2023 Feb 13;3(2):e0001582. doi: 10.1371/journal.pgph.0001582 (PMC10021291; doi:10.1371/journal.pgph.0001582)
Supplement: Attachment — Submitted filename: Response to Editors and Reviewers.doc [file pgph.0001582.s005.doc]

**Response to Editors and Reviewers**

**General response to the reviewers**: We thank the editors and reviewers for their patience with us and having a second look at our paper. We very much appreciate this second set of comments; this has brought us another opportunity to enhance our manuscript. We’ve responded to the comments as follows:

**Comment**: Reviewer #3: The article finds association between VPA / Self-reported health with the social ties and social support. While the authors seem to have addressed most of the comments by previous referee, I have some major concerns regarding the work. The authors have begun with the VPA but have not mentioned whether it is in the form of exercise for health promotion or through employment related reasons. If it is the first, then my concerns are not valid. However, if it is the latter or a mix of both – old age employment (in the informal sector in LMICs) cannot be ignored as it might be driven by lack of formal social security or informal social support and social ties. I feel that this line of reasoning is central to this work which has been not emphasised upon until the discussion section. Therefore, this might require a major rewriting of the paper if I understand it correctly. In addition, there are few minor comments and clarification.

**Our response**: Thank you, and we appreciate your effort to help us improve our manuscript. The older adults included were retired workers, so VPA was measured exclusively in a health promotion context.

**Comment**: (i) People with blindness and mental disorders were excluded through a screening. Please elaborate what screening procedure was used. Kindly include this in the appendix, if appropriate.

**Our response**: Please, the sentence following the selection criteria indicates what was done. A physician assistant screened for those without physical disability at the research site. We have added a sentence to substantiate what was done.

**Comment**: (ii) The concept of negative social capital – does it exist or is it a formulation of authors? In any case this require some elaboration with references.

**Our response**: Thank you. The term has been revised.

**Comment**: (iii) Consider including the survey tool in the appendix for the readers.

**Our response**: Thank you. We’ve added the survey as appendix A.

**Comment:** (iv) Social ties and Social support – Please define which variables that have been collected have been used to explain these concepts. with respect to the variables you have collected.

**Our response**: These variables have been marked out in the survey (appendix A).

**Comment:** (v) Table 1 – Vigorous PA >= 30 code is missing, kindly update. Moreover, table 1 can be considered moving to the appendix.

**Our response**: Very grateful for this; the code is reported now. We’ve also treated this table as Appendix B.

(vi) Pg 13, second para – “…. other than siblings and children mar self-reported health …” Does not make sense – possibly there is some typo.

**Our response**: Thank you. This has been revised.

**Comment:** (vii) Please correct “dependant” that has been used throughout the paper.

**Our response**: Alright, this is done.
