# Supplementary material for: Associations between vigorous physical activity, social ties, social support, and self-reported health among older adults in Accra, Ghana
Source: PLOS Glob Public Health. 2023 Feb 13;3(2):e0001582. doi: 10.1371/journal.pgph.0001582 (PMC10021291; doi:10.1371/journal.pgph.0001582)
Supplement: Attachment — Submitted filename: Response to Editors_R3.doc [file pgph.0001582.s006.doc]

**Response to Editors**

We thank the reviewers and editorial team for their patience and making time to help us improve the manuscript. Your support has been rewarding and we are happy to respond to the additional comments as follows:

Reviewer #5: I commend the persistent effort of the authors with the revision of this article. However, I would like to reiterate the comment of the previous reviewer on the need for clearly describing the key variables as part of the article.

VPA, Social ties, social support etc. are mentioned as key variables in the papers. Since no previously validated tools have been used to assess these variables, a clear description of how these variables have been operationally defined in this study would help the reader with clear understanding.

**Our response**: Please the operational definitions and coding schemes applied to all variables, including the above-listed, are in Appendix B. These were originally provided as a part of the manuscript’s main text, but a reviewer asked for it to be relegated to the appendix. Kindly have a look.

**Comment**: Moreover, the questionnaire (Appendix A) include a section on health behaviors such as smoking, alcohol consumption, duration of watching TV etc. and type of food consumption. But, these variables are missing in the analysis. Consider including these variables in the analysis as they are probable risk factors of health status.

**Our response**: Thanks a great deal for your attention to detail. This has helped us to realize our oversight. The original questionnaire developed contained the above variables (i.e., alcohol consumption, smoking status, etc.), but the research team later revised this into a shorter questionnaire in the light of changes in the research aim and scope. However, both the original questionnaire and revised (shortened) questionnaire were kept in the same project folder. The corresponding author mistakenly submitted the old (original) questionnaire with the above variables, though data were not gathered on them, and they were not part of the current analysis. For these reasons, we have replaced the questionnaire in the appendix with the revised (shorter) questionnaire with which data were generated. In any case, we are very grateful to you for being judicious about the review and helping us to avoid this serious mistake.
