# Supplementary material for: Associations between vigorous physical activity, social ties, social support, and self-reported health among older adults in Accra, Ghana
Source: PLOS Glob Public Health. 2023 Feb 13;3(2):e0001582. doi: 10.1371/journal.pgph.0001582 (PMC10021291; doi:10.1371/journal.pgph.0001582)
Supplement: Attachment — Submitted filename: Response to Reviewers_R4_PLOS Global Health.doc [file pgph.0001582.s007.doc]

**Response to Reviewers**

**Our general response**: We are grateful to the editorial team for the patience they’ve had with our manuscript. We also value their effort to maximise the quality of our manuscript. We accept all the comments and address each as indicated as follows. However, we plead with the reviewers to identify all issues once and for all since they seem to be reporting different issues after our revision.

Reviewer #6: I believe the authors did a good job at addressing the concerns of the reviewers from the previous rounds of reviews.

That said, I have some minor concerns:

1. The statistical method does not mention the criteria for variable selection for the multivariable model.

**Our response**: We’ve reported these criteria under statistical analysis method, though we thought these were reported earlier under measurement.

2. How was collinearity addressed in your adjusted model? I reckon that potential collinearity exists between variables such as dependents vs. children; the number of close friends vs. social support.

**Our response**: to the best of our knowledge, logistic regression, unlikely multiple linear regression, is a non-parametric test, and therefore does not assess collinearity. Even so, we have acknowledged the issue in the limitation.

**Comment:** 3. Did you present the crude odd ratio and adjusted odd ratio in Table 2? It is a bit confusing. I recommend indicating crude odd ratio as 'OR', and Adjusted odd ratio as 'aOR'

**Our response**: Thank you. We’ve revised appropriately. AOR stands for adjusted odds ratio whereas OR stands for crude odds ratio.

4. I could not locate Appendix B.

**Our response**: It was attached as a supplementary material, so please download it as a separate file.

Reviewer #7: The findings section of the manuscript needs grammatical revisions. The missing of words like "were" is making it difficult to understand the message being communicated.

A revision read to correct any grammatical errors in the manuscript can help improve the written quality of the draft especially for the findings section to make it much easier for the people reading to understand the findings of the research.

**Our response**: Thanks. We’ve sent the manuscript for professional proofreading. Kindly check if the revisions are satisfactory.

________________________________________
